# Release of polychlorinated biphenyls (PCBs) and organochlorine pesticides (OCPs) from cigarette butts into the aquatic environment: Levels and ecotoxicity

**DOI:** 10.1016/j.heliyon.2024.e39046

**Published:** 2024-10-09

**Authors:** Hossein Arfaeinia, Mohammad Reza Masjedi, Rasoul Asgariyan, Farshid Soleimani, Vali Alipour, Sara Dadipoor, Reza Saeedi, Anis Jahantigh, Ammar Maryamabadi

**Affiliations:** a Department of Environmental Health Engineering, Faculty of Health, Bushehr University of Medical Sciences, Bushehr, Iran; b Tobacco Control Research Center (TCRC), Iranian Anti-Tobacco Association, Tehran, Iran; c Department of Environmental, Esfahan Steel Company, Esfahan, 8477153111, Iran; d Tobacco and Health Research Center, Hormozgan University of Medical Sciences, Bandar Abbas, Iran; e Department of Health, Safety and Environment (HSE), School of Public Health and Safety, Shahid Beheshti University of Medical Sciences, Tehran, Iran; f Health Promotion Research Center, Zahedan University of Medical Sciences, Zahedan, Iran; g R &D Department, Shakheh Zeytoon Lian Co., Bushehr, Iran

**Keywords:** Cigarette butts, Leachates, Polychlorinated biphenyls, Organochlorine pesticides, Water bodies

## Abstract

Discarded cigarette waste may leach toxic elements and can contaminate different environments. In this study, the levels of organochlorine pesticides (OCPs) and polychlorinated biphenyls (PCBs) in cigarette butts (CBs) leachates were determined, and the release rate of these pollutants from three CBs types such as smoked CBs with and without tobacco (SCBs and SFs) and unsmoked filters (USFs) were examined. The mean concentration levels of PCBs compounds were <LOD-1.36, <LOD-1.02 and <LOD-0.86 μg/L in SCBs, SFs, and USFs leachates, respectively. The concentrations of OCPs in SCBs and SFs leachates were <LOD-0.14 and <LOD-0.10 μg/L, respectively. The toxic equivalent (TEQ) of the dioxin-like PCBs from the CBs leachate samples ranged from 2 × 10^−6^-1 × 10^−4^ ng/L. Notwithstanding the insignificant TEQ values in CBs leachates, continuous discharge and the toxicities of the PCBs in the aquatic environments could have harmfully impacts on the water boddies and/or human health. Globally, considering all the littered CBs and their chemical contents, further efforts are needed to investigate address, and mitigate this type of contamination.

## Highlights

• Three types of cigarette butts leachates were evaluated for PCBs and OCPs.
•PCB105 was the most dominant dioxin-like PCBs compounds.
•PCB194 was the most dominant non-dioxin-like PCBs compounds.
•α-Lindane was observed in all three types cigarette butts leachates.
•The levels of ∑OCPs in three types of leachates were significantly different.

## Graphical abstract

**Figure:**
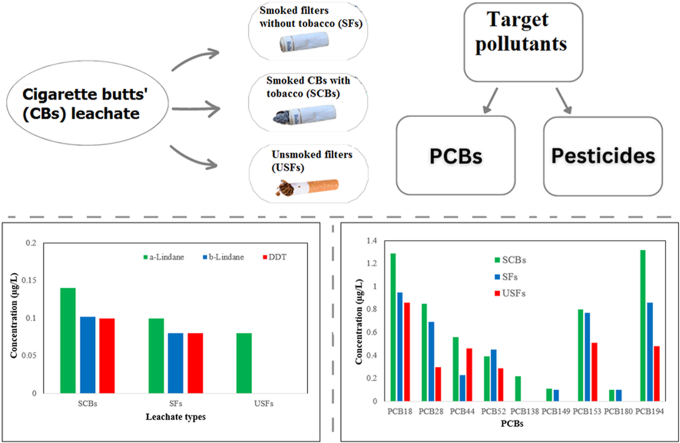

## 1 Introduction

The use of tobacco products is well known as a severe threat to health and the environment [[1], [2], [3], [4], [5], [6], [7]]. Cigarettes are the most frequently used tobacco product and, about 1-in-7 of the people smokes around the world [8], which 75–97.8 % of smokers littered their cigarette butts (CBs) into the surroundings improperly [[9], [10], [11]]. Generally, it has been reported that CBs cover 13 % of the total items collected in the international coastal cleanup [12]. These wastes are present in public places (i.e. bus stations, gardens, roads, and seaboards) [13, 14], and may accumulate along the coastline, gardens, and streets and have ecological and health effects [15]. The discarded CBs could transport to the oceans through storm sewers, runoff, and rivers [16] and remaines suspended in the water body and then reached the floor sediment [17]. In a study, the CB density in the northern Persian Gulf, Iran ranged from 2 to 38 items/m^2^ [18]. Considering the wast range of the littered CBs, the toxic impacts of CBs on aquatic and/or terrestrial organisms have been confirmed previously [[19], [20], [21], [22], [23], [24], [25], [26], [27], [28], [29], [30]]. The toxicity of CBs is associated with the chemical contents of cigarettes. Cigarettes smoke contains over 4000 chemicals [31,32], including polycyclic aromatic hydrocarbons (PAHs) [[33], [34], [35]], nicotine [36], aromatic amines [37], heavy metals [[38], [39], [40], [41], [42], [43], [44]], tobacco-specific nitrosamines [45,46], phenols [45,46], insecticides [[47], [48], [49]], and others. Although numerous chemicals have been detected in CBs leachates, other toxic compounds that are probably exist in CBs, such as herbicides, pesticides, polychlorinated biphenyls (PCBs), and biphenyls have not been well considered.

PCBs and organochlorine pesticides (OCPs) are toxic environmental pollutants [50,51]. PCBs have carcinogenic and teratogenic properties [52,53], and can bioaccumulate in the human body [54]. Previous scientific reports showed the influence of smoking on POPs levels (i.e. PCBs) in humans [[55], [56], [57], [58]]. In a study by Moon et al. (2017), the serum levels of PCB 156, 167, and 180 were pointedly higher in smokers than nonsmokers. These compounds can enter the human body through inhalation and/or skin routes [59]. The volatilization of products containing PCBs in landfills is a predominant source of PCBs in the atmosphere [60,61]. Considering that CBs make up the majority of waste in annual coastal/urban cleanups [62,63], littered CBs are a main source for the release of PCBs. Determination of PCBs in tobacco industry products is essential, and there are limited studies on PCBs contents in these products. PCBs have been observed in unburned [64,65] and mainstream tobacco smoke (MS) [66,67]. OCPs have been generally used to improve agricultural production worldwide [68], which may be used to cultivate of tobacco products. In the case of CBs, the most attention has been paid to the levels of heavy metals and PAHs [34,35,37,[69], [70], [71], [72]], but no attention paid to the levels of OCPs and/or PCBs in CBs. Considering that they are potentially carcinogenic [73], it is necessary to evaluate the concentration of PCBs and OCPs in CBs. This study was aimed to survey the PCBs and OCPs in CBs leachates into the aquatic environment and their ecotoxicity levels for environment. Our hypotheses were that the leached content from CBs may contain OCPs and PCBs in amounts that may be potentially harmful for the environment.

## 2 Method and materials

### 2.1 Stock and sample preparation

In this study, three types of CBs leachates (SCBs: leachate from smoked cigarettes butts with 1.5 cm of residue tobacco, SFs: leachate from smoked cigarettes filters that all residue tobacco removed and, USFs: leachate from unsmoked cigarettes filters) were prepared based on our published studies [30,72]. The Marlboro gold touch brand cigarette as a best-selling brand in Iran, was purchased and machine-smoked at laboratory condition. All cigarettes were smoked at similar conditions [30,72,74]. The stock leachate was prepared based on EPA protocol. CBs were immersed and soaked in natural seawater for a full day [75]. Natural seawater was obtained from the Persian Gulf Research Institute of the Persian Gulf University (seawater was pumped from the sea and filtered through a 1.2 μm pore size, chlorinated, aerated, sand filtered, and stored in tanks). The stock leachate was prepared by adding 4, 8 and,16 CBs to 2 L of natural seawater for SCBs, SFs and, USFs respectively for 24 h. Then, the solutions were filtered to remove the suspended particles.

### 2.2 Reagents and materials

In order to determination of PCBs and OCPs levels, dichloromethane, chromatographic grade water, acetone, acetonitrile and, n-hexane were purchased from Carlo-Erba (Val-de-Reuil, France). All of 13 PCBs (PCB18, PCB28, PCB44, PCB52, PCB77, PCB105, PCB114, PCB118, PCB138, PCB149, PCB153, PCB180 and PCB194), 12 OCPs (α-Lindane, β-Lindane, γ- Lindane, δ-Lindane, Aldrin, Chlordan, Endosulfan, DDE, Dieldrin, DDD, DDT and Metoxychlor) and triphenylphosphate (TPP) were purchased separately from LGC group (Dr. Ehrenstorfer) as single standard solutions.

### 2.3 GC/MS equipment and extraction process

In order to extract the PCBs and OCPs from leachate samples, the sequences of tests were accompanied by taking 10 mL portions from three types of leachates. The dispersive liquid-liquid extraction method (DLLME) was used to the extraction of PCBs. Dichloromethane (DCM) and acetone were used as extraction and dispersion solvents, respectively. Briefly, 10 mL of leachate sample was introduced into a 15 mL polypropylene tube, 50 μL of TPP solution was added and 1 mL of DLLME mixture (Acetone: DCM 9:1) was fast injected into leachate to form cloudy solution. This solution was vortexed for 1 min and centrifuged for 5 min at 6500 rpm. The lower phase (DCM) containing PCBs and OCPs was used for injection and analysis by Agilent 7890GC/5977MSD chromatograph. For each type of leachate, 15 samples were tested. All expriments were performed in 3 repetition [76]. The repetitions were done on the same day to evaluate the precision of the analytical method.

The used GC/MSD was equipped with an ultra inert column (30 m × 0.25 mm × 25 μm) and a split/splitless inlet. The oven temperature was programed as follows: 1 min at 55 °C, temperature ramp of 8 °C min^−1^ up to 150 °C for 2 min and, final ramp of 20 °C min^−1^ up to 280 °C for 15 min. Then, one μL of the extract was injected and uration of pulse was 40 psi for 0.2 min at 300 °C. Selected ion monitoring (SIM) mode was employed to detect and quantify the PCBs and OCPs. One quantifier ion and two qualifiers have been used (Table S1). The recommended standards and relative response factors for OCPs and PCBs quantitation analysis are presented in Table S2.

### 2.4 The quality control/assurance

Beside the tests of PCBs and OCPs in CB leachates, spiked samples were also set from adding the diverse concentrations of the standard samples. Method and spiked blank samples were examined for three days (three times repetition). Indeed, the accuracy and precision of the method were determined by spiking several levels of internal standard (5, 10, and 20 μg/L in CB leachates) into the blank matrix, with three replications for each level. Eventually, tiny levels observed for some congeners of PCBs and OCPs in blanks were adequately subtracted from the values detected in the leachates samples. RSD% was considered as index for accuracy and intraday experiments were considered as indicators for precision. The limit of detection (LOD), and quantitation (LOQ) for each PCBs and OCPs compounds were determined based on a signal-to-noise ratio equal to 3 and 10, respectively. The LOD and LOQ values are presented in Table S2. These values were 0.03–0.07 and 0.10–0.23 μg/L for PCBs and 0.06–0.07 and 0.18–0.26 μg/L for OCPs, respectively. The recovery values were 70.84–99.07 μg/L for PCBs and 74.83–99.51 μg/L for OCPs. The average recoveries lower than 80 % could be considered as poor separation and could be explained as spiking carried out at a very deficient level. Those estimated values for accuracy experiment complied the highest requirements specified for deficient spiking level [77].

### 2.5 Toxicity of DL-PCBs

The toxic equivalent (TEQ) was determined according to Van et al. (2006) study [78]. The TEQ values were determined using the obtained DL-PCB values in different CB leachates and WHO (2005) Toxic Equivalency Factors (TEFs) levels for human and mammals [78] based on equation (1). The toxic equivalency of a mixture is defined by the sum of the concentrations of individual compounds (Ci) multiplied by their relative toxicity (TEF). The TEF values are 0.0001 for PCB 77; 0.00003 for PCB 105, 114, and 118.

Equation 1 TEQ=Σ[Ci×TEFi]

The ecological risk quotient (RQ) was also used to assess the ecological risk of detected OCP compounds in the water media. The formula for calculating the RQ is as follows:

Equation 2 $$\;\text{RQ}=\frac{\text{MEC}}{\text{PNEC}}$$

where MEC is the detected concentration of OCPs (ng/l) leachate samples, and PNEC is the predicted no-effect concentration were acquired from Guo et al. (2023) study [79]. The PNEC values were calculated by dividing the LC_50_ or EC_50_ and the value of Assessment Factors (AF) were considered 1000 for all compounds [79]. The RQ values of 0.1–1 indicate moderate environmental risk and values less than 0.1 indicate low environmental risk [80].

### 2.6 Statistical analyzes

Statistical analyses were done with SPSS (V. 26). The ANOVA and Tukey Post Hoc analyzes were used for determine the significant differences in PCBs and OCPs concentrations among the different CB leachates. The p-value less than 0.05 was considered as statistically significance.

## 3 Results and discussion

### 3.1 PCBs levels in CBs

The mean levels of ∑PCBs in the three types of leachates (SCBs, SFs, and USFs) were <LOD - 1.36, <LOD - 1.02 and, <LOD - 0.86 μg/L, respectively (Table 1). PCB18, PCB28, PCB44, PCB52, PCB77, PCB105, PCB114, PCB149, PCB153 and, PCB194 were identified in all leachate types. The highest values in SCBs, SFs and, USFs leachates were related to PCB105, PCB105 and, PCB18 respectively. The total values of PCBs (∑PCBs) were ranked as: SCBs > SFs > USFs. The ∑PCBs values of SCBs leachate was significantly greater than both SFs and USFs leachates (p-value<0.001). The mean levels of all PCBs (except PCB52, PCB114 and, PCB149) and ∑PCBs in diverse leachates were significantly different (p-value<0.005). The Multiple/Post Hoc comparisons are provided in Table S3.

**Table 1:** The mean (±SD) levels of PCBs (μg/L) in different leachates (n = 15).

PCBs	SCBs	SFs	USFs	p-value
**PCB18**	1.29 ± 0.03	0.95 ± 0.03	0.86 ± 0.02	<0.001
**PCB28**	0.85 ± 0.03	0.69 ± 0.02	0.30 ± 0.01	<0.001
**PCB44**	0.56 ± 0.04	0.23 ± 0.02	0.46 ± 0.03	0.001
**PCB52**	0.39 ± 0.02	0.45 ± 0.03	0.29 ± 0.02	0.129
**PCB77**	1.29 ± 0.05	0.95 ± 0.04	0.51 ± 0.01	<0.001
**PCB105**	1.39 ± 0.07	1.02 ± 0.05	0.78 ± 0.02	0.005
**PCB114**	0.14 ± 0.10	0.09 ± 0.002	0.08 ± 0.006	0.08
**PCB118**	<LOD	0.19 ± 0.02	<LOD	–
**PCB138**	0.22 ± 0.01	<LOD	<LOD	–
**PCB149**	0.11 ± 0.02	0.10 ± 0.008	<LOD	0.215
**PCB153**	0.80 ± 0.04	0.77 ± 0.06	0.51 ± 0.06	0.01
**PCB180**	0.10 ± 0.12	0.10 ± 0.01	<LOD	–
**PCB194**	1.32 ± 0.09	0.86 ± 0.08	0.48 ± 0.02	<0.001
**∑PCBs**	8.43 ± 0.53	6.40 ± 0.39	4.27 ± 0.30	<0.001

**Fig. 1:**
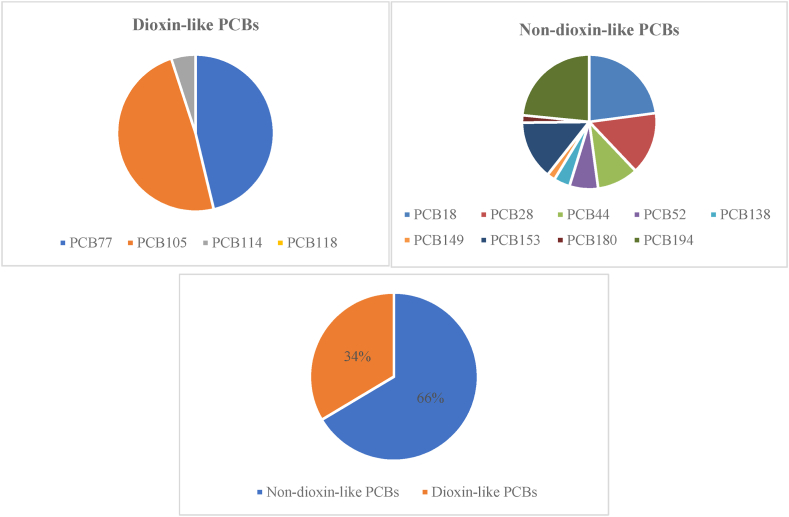
The proportion of Dioxin-like (DL) and Non-Dioxin like (Non-DL) PCBs in CBs leachates.

Some PCBs such as PCB 77, 81, 105, 114, 118, 123, 126, 156, 157, 167, 169, and 189, due to having similar chemical structures with dioxins and furans, as well as harmful effects on health are recognized as dioxin-like PCBs (DL-PCBs) [81,82]. The content of DL and Non-Dioxin like (Non-DL) PCBs in CBs leachates are shown in Fig. 1. As shown, among the dioxin-like PCBs, PCB105, and in the case of Non-DL PCBs, PCB194 were the most dominant compounds. As well as, 34 % of the total PCBs identified in the CBs leachate samples were DL while the Non-DL PCBs has a percentage distribution of 66 %. In Adesina et al. (2022), study aldo 40 % of the total PCBs found in the MS were DL and, the Non-DL PCBs have a percentage distribution of 60 % [66].

In this study, three DL-PCBs (PCB 77, 105 and 114) were detected in all three types of leachates, and PCB118 was only identified in SFs leachates. In order to calculate the possible toxicity of DL-PCBs in CB leachates, the TEQ method was assumed for evaluation the human exposure and health risks of the DL congeners, which contained 34 % of the total PCBs levels in CB leachates. The TEQs of the DL-PCBs in the CB leachates ranged from 2 × 10^−6^-1 × 10^−4^ ng/L (Table 2). Despite the shallow TEQs values in CB leachates, continuous discharge and the toxicities of the PCBs in the aquatic environments could have unfavorably impacts on the water bodies and human health. However, the application of TEF/TEQ approach is primarily for estimating dietary exposure, and the application TEF values (consumption or ingestion) to calculate the TEQ in abiotic environmental matrices and use for risk assessment is limited, unless the aspects of reducing bioavailability, transport and, environmental fate of the intended compounds should be considered. In order to assess the human risk for abiotic matrices, specific homologous equations should be used in the whole model instead of using the total TEQ model, because the fate and transfer between congeners are different [78].

**Table 2:** The toxicity equivalent (TEQ) and Toxic Equivalency Factors (TEFs) values of PCBs.

PCBs	TEF	TEQ_SCBs_ (Ci ∗ TEF)	TEQ_SFs_ (Ci ∗ TEF)	TEQ_USFs_ (Ci ∗ TEF)
**PCB77**	0.0001	1E-04	1E-04	5E-05
**PCB105**	0.00003	4E-05	3E-05	2E-05
**PCB114**	0.00003	4E-06	3E-06	2E-06
**PCB118**	0.00003	–	6E-06	–

The toxicity of CB leachates to aquatic and/or terrestria organisms has been well proven in recent studies. The existing data show that CB leachates are toxic to many organisms, in many parts of the world [[28], [29], [30],[83], [84], [85]], and different survival, growth, and reproduction effects on aquatic organism are reported in a systematic review [29]. For example, the results of Montalvao et al. (2019) study showed that CB leachates causes mutagenic effect in representatives of a freshwater bivalve group [27,83]. Also, Parker & Rayburn reported that CBs leachate have teratogenic impact to Xenopus laevis embryos [86]. In another study DNA damage in ragworms exposed to SCB was >2-fold increase in compared to control group [87]. These results clearly reveal the potential toxicity of CBs for aquatic organisms and their fate in aquatic environments. Although the mechanism and intensity of toxicity of CBs on aquatic organisms are complex and are among the limitations of recent studies, it is recommended to carry out new researchs on the mechanism, intensity and range of effects on different organisms.

### 3.2 The OCPs levels in CBs leachates

**Fig. 2:**
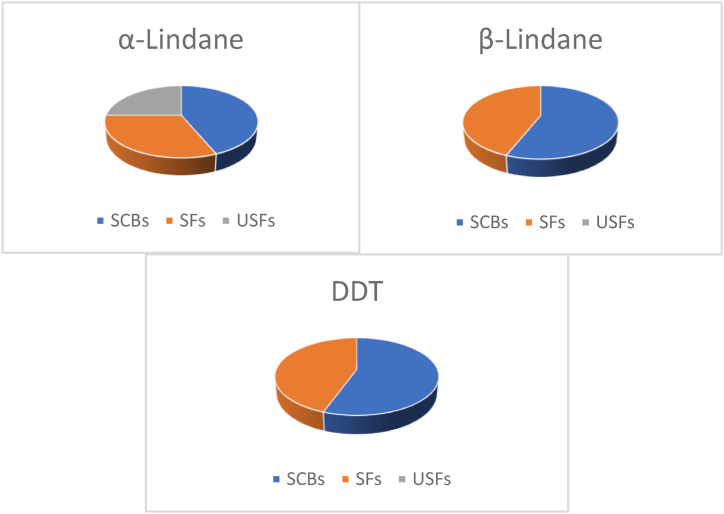
The levels of unique OCPs in all three CB leachate types (μg/L).

The mean concentrations of OCPs (μg/L) in SCBs and SFs leachates ranged from <LOD to 0.14 and <LOD to 0.10 μg/L, respectively. In the case of USFs leachates, these values were <LOD for all compounds except α-Lindane with 0.08 μg/L (Table 3 and Fig. 2). α-Lindane was observed in all three SCBs, SFs and, USFs leachates. The mean concentrations of α- Lindane and DDT in different leachate types were differed significantly (p-value<0.005).

**Table 3:** The mean levels of OCPs (μg/L) in various CB leachate types (n = 15).

OCPs	SCBs	SFs	USFs	p-value
**α-Lindane**	0.14 ± 0.001	0.10 ± 0.001	0.08 ± 0.004	0.001
**β-Lindane**	0.10 ± 0.002	0.08 ± 0.0005	<LOD	–
**γ-Lindane**	<LOD	<LOD	<LOD	–
**δ-Lindane**	0.11 ± 0.001	<LOD	<LOD	–
**Aldrin**	<LOD	<LOD	<LOD	–
**Chlordan**	<LOD	<LOD	<LOD	–
**Endosulfan**	<LOD	<LOD	<LOD	–
**DDE**	<LOD	<LOD	<LOD	–
**Dieldrin**	<LOD	<LOD	<LOD	–
**DDD**	<LOD	<LOD	<LOD	–
**DDT**	0.10 ± 0.001	0.08 ± 0.001	<LOD	0.045
**Metoxychlor**	<LOD	<LOD	<LOD	–

Previous relationships have been observed between smoking and POPs, PCBs and/or OCPs on mortality [88,89]. In a study by Quadroni and Bettinetti (2019), the presence of DDT and lindane pesticides in tobacco products were assessed and in 78.57 % of samples, Dichlorodiphenyltrichloroethane (DDTs) were the dominant with nine ng/g pp'-DDT and 13 ng/g pp'-DDE. The α/γ-HCH ratio was also mostly <1, demonstrating the highest use of "lindane" formulation [90]. In Rahman et al. (2012) study, the mean value of DDT residues was 4000 ng/g in tobacco leaf samples collected from Bangladesh [91]. Asubiojo et al. (2009) detected the DDT metabolite (pp’DDD) in Nigeria cigarette samples [92]. However, Hristova-Bagdassarian et al. (2009) did not detect DDTs and HCHs chemicals in tobacco leave samples from Bulgaria [93]. DDT and lindane are two organochlorine compounds that are still illegally in use for ensuring tobacco crops production in a wide range [90].

The ecological risk of OCPs in water media are presended in Table 4. The ecological risk assessment of single OCPs showed that the RQ values of α-Lindane and DDT in SCB leachates were 0.38 and 3.33 and generally greater than 0.1, with medium and high ecological risks respectively. The RQ values of α-Lindane and DDT in SF leachates were 0.27 and 2.67 and generally greater than 0.1, with medium and high ecological risks respectively. In the case of USF leachates, the RQ values of α-Lindane (the only detected compound) was 0.22, indicating medium ecological risks.

**Table 4:** Ecological risk parameters associated with organochlorine pesticides (OCPs) in the leachate from various cigarette butts for aquatic media.

**OCPs**	MEC			PNEC	RQ		
	SCB	SF	USF		SCB	SF	USF
α-Lindane	140	100	80	370	0.38	0.27	0.22
β-Lindane	100	80	<LOD	3140	0.032	0.025	
γ-Lindane	<LOD	<LOD	<LOD	2.9			
δ-Lindane	110	<LOD	<LOD	1580	0.07		
Aldrin	<LOD	<LOD	<LOD	10			
Chlordan	<LOD	<LOD	<LOD	90			
Endosulfan	<LOD	<LOD	<LOD	2			
DDE	<LOD	<LOD	<LOD	15			
Dieldrin	<LOD	<LOD	<LOD	50			
DDD	<LOD	<LOD	<LOD	9			
DDT	100	80	<LOD	30	3.33	2.67	
Metoxychlor	<LOD	<LOD	<LOD	150			

The primary sources of contamination of CBs to PCBs and OCPs is tobacco cultivation and processing of products. These contaminants may be add through pesticides application. Pesticides may stay on tobacco leafs after cultivation and processing stages [94]. When cigarette is smoked, its chemical content are burnt, and trapped in the filter. Cigarette combustion temperature degrades chemicals but does not destroy them in tobacco [94]. However, the volatilization temperature of the different chemicals during smoking is not similar and, depends on the boiling and degradation temperature [95]. The transfer ratio of pesticides in tobacco during the smoking into the different parts such as smoke and filter are different [96]. The OCPs transfer ratio from tobacco into MS is approximately 12 % of its amount before burning, and can reach up to 20 and 40 % for DDT and lindane, respectively [94,97]. In a study, the mean level of total pesticide transfer rate into cigarette smoke was 17 % [96]. The pesticide retention for different cellulose filters was 21–40 % [96]. In a study, the transferred pesticides ratio from tobacco into smoke was about 12 % of its amount before burning [94]. Previous studies have also reported up to 20 % of the residue DDT in the unsmoked tobacco [94,98]. In a study by Dávila et al. (2020), the 54.7 % residues of thiodicarb, alachlor, and endosulfan in tobacco smoke were quantifiable and the recovery of these chemicals was higher in the MS than sidestream smoke (SS) and filter [99].

The mean concentrations of α-Lindane and DDT were more significant than other OCP compounds. Regarding available evidence of lindane and DDT carcinogenicity in humans, the IARC categorized these chemicals as carcinogen and possibly carcinogen, respectively [90]. DDT and lindane, as well as their relative metabolites and or isomers are in the list of chemicals introduced by the CORESTA (Cooperation Centre for Scientific Research Relative to Tobacco) [90]. Despite the various chemical compounds in CBs, these wastes are known as hazardous wastes [100]. Considering the significant number of littered CBs, toxicity [29,30,72], as well as different types of chemicals within them [32,72], there should be a particular focus on this global problem. According to these statements, it is really required to find and present a proper method for management the CB litters, because landfill method and/or incineration process cannot be simply used for these litters [100].

## 4 Conclusion

In this research, the levels of OCPs and PCBs were quantified in CB leachates, and the release rate of these contaminats from three CB types including smoked CBs with and without tobacco (SCBs and SFs) and unsmoked filters (USFs) were evaluated. Findings indicated the higher levels of ∑PCBs in SCBs leaching solution compated to both SFs and USFs leachates. As well as, a higher number of OCPs were observed in the SCBs leachates. The considerable differences between PCBs levels in SCBs and SFs leachates may be associated with residues in tobacco which get retained in the SCBs leachates. Although the calculated toxicity values for PCB compounds in CB leachates were not significant, the leaching of these compounds along with other dangerous pollutants from CBs into the water bodies, can bio-accumulate along the food web, causing primary global concern for the environment and affecting the human health and aquatic ecosystem. Further studies are necessary to understand the fate of CBs contaminants and their ecotoxicological effects. As well as, focus on interventions for changing smokers’ awareness and attitude about the health and environmental risks of CBs may be a suitable approach in reducing the amount of these wastes in environment. Future studies are highly recommended to find a appropriate method to safely dispose the safe disposal of these toxic wastes.

## CRediT authorship contribution statement

**Hossein Arfaeinia:** Writing – original draft, Resources, Methodology, Investigation, Formal analysis, Data curation. **Mohammad Reza Masjedi:** Writing – original draft. **Rasoul Asgariyan:** Writing – review & editing, Data curation. **Farshid Soleimani:** Writing – review & editing, Validation, Supervision, Project administration, Methodology, Investigation, Formal analysis, Data curation. **Vali Alipour:** Writing – review & editing, Software. **Sara Dadipoor:** Writing – review & editing, Resources. **Reza Saeedi:** Writing – review & editing, Methodology. **Anis Jahantigh:** Writing – review & editing, Investigation. **Ammar Maryamabadi:** Writing – review & editing, Validation, Formal analysis.

## Ethics approval and consent to participate

“Not applicable”.

## Availability of data and materials

Additional data from the study are available by request to the corresponding author by email.

## Declaration of competing interest

The authors declare that they have no known competing financial interests or personal relationships that could have appeared to influence the work reported in this paper.
