# Supplementary material for: Release of polychlorinated biphenyls (PCBs) and organochlorine pesticides (OCPs) from cigarette butts into the aquatic environment: Levels and ecotoxicity
Source: Heliyon. 2024 Oct 9;10(20):e39046. doi: 10.1016/j.heliyon.2024.e39046 (PMC11620112; doi:10.1016/j.heliyon.2024.e39046)
Supplement: Multimedia component 1 [file mmc1.docx]

**Release of polychlorinated biphenyls (PCBs) and organochlorine pesticides (OCPs) from cigarette butts into the aquatic environment: Levels and ecotoxicity**

**Hossein Arfaeinia^1,†^, Mohammad Reza Masjedi^2, †^, Rasoul Asgariyan^3^, Farshid Soleimani^4,*^, Vali Alipour^4^, Sara Dadipoor^4^, Reza Saeedi^5^, Anis Jahantigh^6^, Ammar Maryamabadi^7^**

^1^Department of Environmental Health Engineering, Faculty of Health, Bushehr University of Medical Sciences, Bushehr, Iran

^2^Tobacco Control Research Center (TCRC), Iranian Anti-Tobacco Association, Tehran, Iran

^3^Department of Environmental, Esfahan Steel Company, Esfahan 8477153111, Iran

^4^Tobacco and Health Research Center, Hormozgan University of Medical Sciences, Bandar Abbas, Iran

^5^Department of Health, Safety and Environment (HSE), School of Public Health and Safety, Shahid Beheshti University of Medical Sciences, Tehran, Iran

^6^Health Promotion Research Center, Zahedan University of Medical Sciences, Zahedan, Iran

^7^R &D Department, Shakheh Zeytoon Lian Co., Bushehr, Iran

*† HA and MRM should be considered as joint first authors*

***Corresponding author**

**Farshid Soleimani**

Tobacco and Health Research Center, Hormozgan University of Medical Sciences, Bandar Abbas, Iran

E-mail address: F.soleimani72@yahoo.com

**Content**

**Table S1**. Selected ions for monitoring PCBs and OCPs

**Table S2**. Recommended standards and relative response factors for OCPs and PCBs quantitation analysis

**Table S3**: Multiple/Post Hoc group comparisons of PCBs (µg/L) in SCBs, SFs and USFs leachates

**Table S1**. Selected ions for monitoring PCBs and OCPs

| **Item** | **R.T** | **Compound** | **m1** | **m2** | **m3** |
| --- | --- | --- | --- | --- | --- |
| **OCPs** | | | | | |
| 1 | 13.87 | a-Lindane | 181 | 183 | 219 |
| 2 | 15.01 | b-Lindane | 181 | 183 | 219 |
| 3 | 15.35 | c-Lindane | 181 | 183 | 109 |
| 4 | 16.78 | d-Lindane | 183 | 181 | 219 |
| 5 | 20.74 | Aldrin | 263 | 261 | 265 |
| 6 | 24.11 | Chlordan | 373 | 375 | 237 |
| 7 | 24.64 | Endosulfan | 207 | 237 | 169 |
| 8 | 25.77 | DDE | 246 | 248 | 210 |
| 9 | 25.81 | Dieldrin | 263 | 265 | 108 |
| 10 | 27.29 | DDD | 235 | 237 | 165 |
| 11 | 28.48 | DDT | 235 | 237 | 165 |
| 12 | 30.21 | Metoxychlor | 227 | 228 | 152 |
| **PCBs** | | | | | |
| 13 | 9.23 | PCB18 | 256 | 258 | 186 |
| 14 | 11.89 | PCB 28 | 186 | 256 | 258 |
| 15 | 12.55 | PCB 52 | 220 | 255 | 292 |
| 16 | 13.00 | PCB 44 | 220 | 222 | 292 |
| 17 | 15.84 | PCB 77 | 110 | 220 | 292 |
| 18 | 16.35 | PCB149 | 360 | 362 | 290 |
| 19 | 16.67 | PCB 118 | 128 | 254 | 326 |
| 20 | 17.09 | PCB 114 | 127 | 184 | 326 |
| 21 | 17.50 | PCB 153 | 145 | 290 | 360 |
| 22 | 17.70 | PCB 105 | 184 | 254 | 326 |
| 23 | 18.69 | PCB 138 | 145 | 290 | 360 |
| 24 | 22.19 | PCB 180 | 324 | 359 | 394 |

**Table S2**. Recommended standards and relative response factors for OCPs and PCBs quantitation analysis

| **Chemicals** | **Retention Time** | **Spiked Concentration (ppb)** | **Average of Obtained Concentration (ppb)** | **Average of Spike Recovery (%)** | **Acceptable range for Spike recovery (%)** | **Intraday RSD% of Spike Recovery** | **Acceptable range for Intraday RSD (%)** | **Interday RSD% of Spike Recovery** | **Acceptable range for Interday RSD (%)** | **LOD (ppb)** | **LOQ (ppb)** |
| --- | --- | --- | --- | --- | --- | --- | --- | --- | --- | --- | --- |
|  |  |  |  |  |  |  |  |  |  |  |  |
|  |  |  |  |  |  |  |  |  |  |  |  |
| a-Lindane | 13.87 | 2 | 1.85 | 92.48 | 60-115 (González and Herrador, 2007) | 7.84 | 21 (González and Herrador, 2007) | 23.95 | 32 (González and Herrador, 2007) | 0.06 | 0.18 |
| b-Lindane | 15.01 |  | 1.45 | 72.58 |  | 7.36 |  | 15.35 |  | 0.06 | 0.18 |
| c-Lindane | 15.35 |  | 1.87 | 93.41 |  | 14.75 |  | 18.14 |  | 0.06 | 0.18 |
| d-Lindane | 16.78 |  | 1.89 | 94.55 |  | 14.65 |  | 23.99 |  | 0.06 | 0.18 |
| Aldrin | 20.74 |  | 1.53 | 76.54 |  | 10.37 |  | 15.78 |  | 0.08 | 0.26 |
| Chlordan | 24.11 |  | 1.88 | 94.18 |  | 12.07 |  | 21.70 |  | 0.07 | 0.24 |
| Endosulfan | 24.64 |  | 1.91 | 95.70 |  | 14.24 |  | 18.80 |  | 0.07 | 0.23 |
| DDE | 25.77 |  | 1.42 | 70.84 |  | 11.27 |  | 19.63 |  | 0.06 | 0.21 |
| Dieldrin | 25.81 |  | 1.98 | 99.07 |  | 16.50 |  | 15.36 |  | 0.07 | 0.25 |
| DDD | 27.29 |  | 1.78 | 88.80 |  | 7.79 |  | 18.27 |  | 0.07 | 0.22 |
| DDT | 28.48 |  | 1.49 | 74.73 |  | 13.67 |  | 20.95 |  | 0.06 | 0.21 |
| Metoxychlor | 30.21 |  | 1.52 | 76.10 |  | 13.49 |  | 20.14 |  | 0.07 | 0.24 |
| PCB18 | 9.23 |  | 1.97 | 98.35 |  | 7.91 |  | 21.24 |  | 0.04 | 0.12 |
| PCB 28 | 11.89 |  | 1.99 | 99.51 |  | 13.43 |  | 16.65 |  | 0.04 | 0.12 |
| PCB 52 | 12.55 |  | 1.91 | 95.53 |  | 16.38 |  | 22.63 |  | 0.03 | 0.11 |
| PCB 44 | 13 |  | 1.97 | 98.55 |  | 7.07 |  | 15.19 |  | 0.03 | 0.10 |
| PCB 77 | 15.84 |  | 1.97 | 98.63 |  | 7.35 |  | 20.91 |  | 0.04 | 0.14 |
| PCB149 | 16.35 |  | 1.72 | 86.01 |  | 11.74 |  | 20.75 |  | 0.05 | 0.16 |
| PCB 118 | 16.67 |  | 1.91 | 95.41 |  | 13.66 |  | 22.32 |  | 0.04 | 0.12 |
| PCB 114 | 17.09 |  | 1.62 | 81.10 |  | 11.06 |  | 19.80 |  | 0.04 | 0.14 |
| PCB 153 | 17.5 |  | 1.80 | 90.06 |  | 15.81 |  | 22.16 |  | 0.05 | 0.16 |
| PCB 105 | 17.7 |  | 1.67 | 83.73 |  | 7.91 |  | 20.57 |  | 0.04 | 0.14 |
| PCB 138 | 18.69 |  | 1.73 | 86.41 |  | 11.83 |  | 18.79 |  | 0.06 | 0.21 |
| PCB 180 | 22.19 |  | 1.50 | 74.83 |  | 11.31 |  | 16.47 |  | 0.07 | 0.23 |

**Table S3**. Multiple/Post Hoc group comparisons of PCBs^a^ (µg/L) in SCBs^b^, SFs^c^ and USFs^d^ leachates

| Dependent Variable | (I) group | (J) group | Mean Difference (I-J) | Std. Error | Sig. | 95% Confidence Interval | |
| --- | --- | --- | --- | --- | --- | --- | --- |
|  |  |  |  |  |  | Lower Bound | Upper Bound |
| PCB18 | SCBs | SFs | .34000^*^ | .04472 | .000 | .2306 | .4494 |
|  |  | USFs | .43000^*^ | .04472 | .000 | .3206 | .5394 |
|  | SFs | SCBs | -.34000^*^ | .04472 | .000 | -.4494 | -.2306 |
|  |  | USFs | .09000 | .04472 | .091 | -.0194 | .1994 |
|  | USFs | SCBs | -.43000^*^ | .04472 | .000 | -.5394 | -.3206 |
|  |  | SFs | -.09000 | .04472 | .091 | -.1994 | .0194 |
| PCB28 | SCBs | SFs | .16000^*^ | .03367 | .003 | .0776 | .2424 |
|  |  | USFs | .55000^*^ | .03367 | .000 | .4676 | .6324 |
|  | SFs | SCBs | -.16000^*^ | .03367 | .003 | -.2424 | -.0776 |
|  |  | USFs | .39000^*^ | .03367 | .000 | .3076 | .4724 |
|  | USFs | SCBs | -.55000^*^ | .03367 | .000 | -.6324 | -.4676 |
|  |  | SFs | -.39000^*^ | .03367 | .000 | -.4724 | -.3076 |
| PCB44 | SCBs | SFs | .33333^*^ | .00981 | .000 | .3093 | .3573 |
|  |  | USFs | .09000^*^ | .00981 | .000 | .0660 | .1140 |
|  | SFs | SCBs | -.33333^*^ | .00981 | .000 | -.3573 | -.3093 |
|  |  | USFs | -.24333^*^ | .00981 | .000 | -.2673 | -.2193 |
|  | USFs | SCBs | -.09000^*^ | .00981 | .000 | -.1140 | -.0660 |
|  |  | SFs | .24333^*^ | .00981 | .000 | .2193 | .2673 |
| PCB52 | SCBs | SFs | -.06000^*^ | .02449 | .050 | -.1199 | -.0001 |
|  |  | USFs | .10000^*^ | .02449 | .006 | .0401 | .1599 |
|  | SFs | SCBs | .06000^*^ | .02449 | .050 | .0001 | .1199 |
|  |  | USFs | .16000^*^ | .02449 | .001 | .1001 | .2199 |
|  | USFs | SCBs | -.10000^*^ | .02449 | .006 | -.1599 | -.0401 |
|  |  | SFs | -.16000^*^ | .02449 | .001 | -.2199 | -.1001 |
| PCB77 | SCBs | SFs | .34000^*^ | .03692 | .000 | .2497 | .4303 |
|  |  | USFs | .78667^*^ | .03692 | .000 | .6963 | .8770 |
|  | SFs | SCBs | -.34000^*^ | .03692 | .000 | -.4303 | -.2497 |
|  |  | USFs | .44667^*^ | .03692 | .000 | .3563 | .5370 |
|  | USFs | SCBs | -.78667^*^ | .03692 | .000 | -.8770 | -.6963 |
|  |  | SFs | -.44667^*^ | .03692 | .000 | -.5370 | -.3563 |
| PCB105 | SCBs | SFs | .36000^*^ | .01414 | .000 | .3254 | .3946 |
|  |  | USFs | .58000^*^ | .01414 | .000 | .5454 | .6146 |
|  | SFs | SCBs | -.36000^*^ | .01414 | .000 | -.3946 | -.3254 |
|  |  | USFs | .22000^*^ | .01414 | .000 | .1854 | .2546 |
|  | USFs | SCBs | -.58000^*^ | .01414 | .000 | -.6146 | -.5454 |
|  |  | SFs | -.22000^*^ | .01414 | .000 | -.2546 | -.1854 |
| PCB114 | SCBs | SFs | .06000 | 2.15694 | .979 | -5.2178 | 5.3378 |
|  |  | USFs | -2.57667 | 2.15694 | .277 | -7.8545 | 2.7012 |
|  | SFs | SCBs | -.06000 | 2.15694 | .979 | -5.3378 | 5.2178 |
|  |  | USFs | -2.63667 | 2.15694 | .267 | -7.9145 | 2.6412 |
|  | USFs | SCBs | 2.57667 | 2.15694 | .277 | -2.7012 | 7.8545 |
|  |  | SFs | 2.63667 | 2.15694 | .267 | -2.6412 | 7.9145 |
| PCB118 | SCBs | SFs | -.19000^*^ | .00471 | .000 | -.2015 | -.1785 |
|  |  | USFs | .00000 | .00471 | 1.000 | -.0115 | .0115 |
|  | SFs | SCBs | .19000^*^ | .00471 | .000 | .1785 | .2015 |
|  |  | USFs | .19000^*^ | .00471 | .000 | .1785 | .2015 |
|  | USFs | SCBs | .00000 | .00471 | 1.000 | -.0115 | .0115 |
|  |  | SFs | -.19000^*^ | .00471 | .000 | -.2015 | -.1785 |
| PCB138 | SCBs | SFs | .22000^*^ | .00943 | .000 | .1969 | .2431 |
|  |  | USFs | .22000^*^ | .00943 | .000 | .1969 | .2431 |
|  | SFs | SCBs | -.22000^*^ | .00943 | .000 | -.2431 | -.1969 |
|  |  | USFs | .00000 | .00943 | 1.000 | -.0231 | .0231 |
|  | USFs | SCBs | -.22000^*^ | .00943 | .000 | -.2431 | -.1969 |
|  |  | SFs | .00000 | .00943 | 1.000 | -.0231 | .0231 |
| PCB149 | SCBs | SFs | .00333 | .00385 | .420 | -.0061 | .0128 |
|  |  | USFs | .10667^*^ | .00385 | .000 | .0972 | .1161 |
|  | SFs | SCBs | -.00333 | .00385 | .420 | -.0128 | .0061 |
|  |  | USFs | .10333^*^ | .00385 | .000 | .0939 | .1128 |
|  | USFs | SCBs | -.10667^*^ | .00385 | .000 | -.1161 | -.0972 |
|  |  | SFs | -.10333^*^ | .00385 | .000 | -.1128 | -.0939 |
| PCB153 | SCBs | SFs | .02333 | .02762 | .431 | -.0443 | .0909 |
|  |  | USFs | .80000^*^ | .02762 | .000 | .7324 | .8676 |
|  | SFs | SCBs | -.02333 | .02762 | .431 | -.0909 | .0443 |
|  |  | USFs | .77667^*^ | .02762 | .000 | .7091 | .8443 |
|  | USFs | SCBs | -.80000^*^ | .02762 | .000 | -.8676 | -.7324 |
|  |  | SFs | -.77667^*^ | .02762 | .000 | -.8443 | -.7091 |
| PCB180 | SCBs | SFs | -.21333 | .15926 | .229 | -.6030 | .1764 |
|  |  | USFs | .09667 | .15926 | .566 | -.2930 | .4864 |
|  | SFs | SCBs | .21333 | .15926 | .229 | -.1764 | .6030 |
|  |  | USFs | .31000 | .15926 | .100 | -.0797 | .6997 |
|  | USFs | SCBs | -.09667 | .15926 | .566 | -.4864 | .2930 |
|  |  | SFs | -.31000 | .15926 | .100 | -.6997 | .0797 |
| PCB194 | SCBs | SFs | .44667^*^ | .02419 | .000 | .3875 | .5059 |
|  |  | USFs | 1.32000^*^ | .02419 | .000 | 1.2608 | 1.3792 |
|  | SFs | SCBs | -.44667^*^ | .02419 | .000 | -.5059 | -.3875 |
|  |  | USFs | .87333^*^ | .02419 | .000 | .8141 | .9325 |
|  | USFs | SCBs | -1.32000^*^ | .02419 | .000 | -1.3792 | -1.2608 |
|  |  | SFs | -.87333^*^ | .02419 | .000 | -.9325 | -.8141 |
| ∑PCBs | SCBs | SFs | 2.05000^*^ | .07808 | .000 | 1.8589 | 2.2411 |
|  |  | USFs | 4.17667^*^ | .07808 | .000 | 3.9856 | 4.3677 |
|  | SFs | SCBs | -2.05000^*^ | .07808 | .000 | -2.2411 | -1.8589 |
|  |  | USFs | 2.12667^*^ | .07808 | .000 | 1.9356 | 2.3177 |
|  | USFs | SCBs | -4.17667^*^ | .07808 | .000 | -4.3677 | -3.9856 |
|  |  | SFs | -2.12667^*^ | .07808 | .000 | -2.3177 | -1.9356 |
| *. The mean difference is significant at the 0.05 level. | | | | | | | |

^a^PCBs: Polychlorinated biphenyls

^b^SCBs: Smoked cigarette butts with tobacco

^c^SFs: Smoked filters without tobacco

^d^USFs: Unsmoked filters
